# Optimizing the data combination rule for seamless phase II/III clinical trials

**DOI:** 10.1002/sim.6316

**Published:** 2014-10-15

**Authors:** Lisa V Hampson, Christopher Jennison

**Affiliations:** a Medical and Pharmaceutical Statistics Research Unit, Department of Mathematics and Statistics, Lancaster UniversityLancaster, U.K.; b Department of Mathematical Sciences, University of BathBath, U.K.

**Keywords:** Bayes decision problem, combination test, closed testing procedure, multiple hypothesis testing, seamless phase II/III trial, treatment selection

## Abstract

We consider seamless phase II/III clinical trials that compare *K* treatments with a common control in phase II then test the most promising treatment against control in phase III. The final hypothesis test for the selected treatment can use data from both phases, subject to controlling the familywise type I error rate. We show that the choice of method for conducting the final hypothesis test has a substantial impact on the power to demonstrate that an effective treatment is superior to control. To understand these differences in power, we derive decision rules maximizing power for particular configurations of treatment effects. A rule with such an optimal frequentist property is found as the solution to a multivariate Bayes decision problem. The optimal rules that we derive depend on the assumed configuration of treatment means. However, we are able to identify two decision rules with robust efficiency: a rule using a weighted average of the phase II and phase III data on the selected treatment and control, and a closed testing procedure using an inverse normal combination rule and a Dunnett test for intersection hypotheses. For the first of these rules, we find the optimal division of a given total sample size between phases II and III. We also assess the value of using phase II data in the final analysis and find that for many plausible scenarios, between 50% and 70% of the phase II numbers on the selected treatment and control would need to be added to the phase III sample size in order to achieve the same increase in power.

## 1 Introduction

In the traditional framework for drug development, a phase II clinical trial compares several doses or formulations of a new treatment against a control. The most promising of these, in terms of efficacy, safety and possibly other considerations, is taken forward to phase III where investigators hope to confirm the benefits of the new treatment in one or two ‘pivotal’ clinical trials.

There has been significant recent interest in combining these two stages of the development process. If a trial follows a ‘seamless’ design, merging the usual phase II and phase III components, there is opportunity to gain additional value from the phase II data by using these together with phase III data in the final analysis. Regulators are liable to treat a combined phase II/III trial as a single study and require a complete protocol to be specified at the outset. This allows a monitoring committee to respond to results on all aspects of the treatments and patient responses at interim points during the trial without further input from the sponsors, who remain blinded to interim results. Seamless designs can be complex, and substantial effort may be required to plan their smooth conduct and establish the validity of the proposed analysis. Thus, it is important that the gains from using phase II data in the final analysis justify this investment.

A variety of methods is available to combine data from the two stages of a seamless design with proper protection of the type I error probability. Thall *et al*. 1 propose two-stage designs with treatment selection at the interim analysis. Sampson and Sill 2 derive most powerful procedures within a certain class of tests combining data from two stages. Bretz *et al*. 3 and Schmidli *et al*. 4 present seamless phase II/III designs that use closed testing procedures 5 to control the familywise type I error rate and combination tests 6 to combine data from the two stages in the final hypothesis test.

It is not obvious how to choose between the various options for combining data across two phases of a seamless trial. Our aims are to identify efficient ways of doing this and, hence, quantify the potential benefits of using phase II data in a final combined analysis. We shall show how to derive an optimal final decision rule, maximizing the probability of selecting the best treatment and declaring it efficacious, under a particular configuration of treatment effects. In some cases, the decision rules we derive only control the familywise error rate over part of the parameter space—but they are still useful as they provide an upper bound on the attainable power and this can be enough to show that certain decision rules, which do control the familywise error rate, are very close to optimal.

In view of the high dimensionality of the parameter space, one would not expect a single data combination rule to be optimal for all parameter vectors. Nevertheless, we have found rules with robust efficiency across a wide range of scenarios. Having an efficient final decision rule is an important pre-requisite for investigating other aspects of phase II/III designs: given such a rule, one can optimize the division of resources between phases or assess the benefits of other phase II options, such as response adaptive allocation of patients to treatments.

In section 2 and section 4 and compare their power functions. In section 5, we derive optimal decision rules for particular configurations of treatment effects: the form of the optimal rules in Section 5.1 helps explain the rather surprising results seen in section 4, and we solve more general optimization problems in Sections 5.2 and  section 6, we compare final decision rules across a range of parameter configurations and identify rules that are highly efficient across a wide variety of situations. In section 7, we focus on one of these robustly efficient rules and show how to determine the most efficient division of resources between phases II and III. In section 8, we assess the benefits of using phase II data in the final decision by computing the number of additional phase III observations that would be needed to produce the same improvement in power. We conclude with a discussion of the implications of our results to extensions of the seamless phase II/III design that we have considered.

## 2 Problem formulation

Henceforth, we shall refer to the two parts of a seamless phase II/III design as stage 1 and stage 2. We consider the format of Thall *et al*. 1 in which *K* experimental treatments are compared with a control in stage 1 and one of these is selected to be tested against the control in stage 2.

We suppose patient responses are normally distributed with known variance *σ*^2^ and means *μ*_0_ on the control arm and *μ*_*i*_, *i* = 1,…,*K*, on treatment arms, with a high mean indicating a successful treatment. We assume that the primary endpoint, the study population and the treatment definitions remain unchanged throughout the trial, so the response distribution for a given treatment in stage 2 is the same as in stage 1. The treatment effects are *θ*_*i*_=*μ*_*i*_−*μ*_0_, *i* = 1,…,*K*, and for now, we make no assumptions about the structure of the vector ***θ*** = (*θ*_1_,…,*θ*_*K*_). There are *K* one-sided null hypotheses *H*_0,1_: *θ*_1_≤0, …, *H*_0,*K*_: *θ*_*K*_≤0 that may be tested, depending on which treatment is selected at the end of stage 1.

Following Thall *et al*. 1 (hereafter TSE), we proceed as follows:

*In stage 1*, randomize *m*_1_ patients to each treatment *i* = 1,…,*K* and the control arm and calculate maximum likelihood estimates 
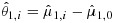
, *i* = 1,…,*K*, of the *K* treatment effects. Let *i*⋆ denote the treatment with maximum 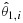. If

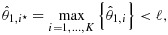
1stop the trial for futility, rejecting no null hypotheses. Otherwise, continue to stage 2 selecting treatment *i*⋆ for comparison with the control.
*In stage 2*, randomize *m*_2_ patients to each of treatment *i*⋆ and the control. Denote the estimate of 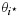
based on stage 2 data only by 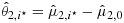.
*In the final analysis,* reject *H*_0,*i*⋆_: 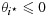 in favour of 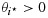 if

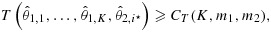
2where the function *T* and critical value *C*_*T*_(*K*,*m*_1_,*m*_2_) are pre-specified.

The familywise error rate (FWER) under ***θ*** of such a procedure is defined as pr{reject any true *H*_0,*i*_;***θ***}. We shall consider procedures that control the FWER strongly at level *α*; that is, they have the property

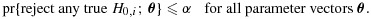

Let *i*_max_ be the index of the treatment with the highest effect *θ*_*i*_. Under parameter vectors ***θ*** for which *i*_max_ is unique and 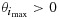, we define the power of a procedure to be

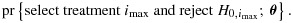
3
Methods of data combination differ in the definition of the function *T* in (2) and the associated critical value *C*_*T*_(*K*,*m*_1_,*m*_2_). Our aims are to compare the power of different final decision rules and identify those with close to optimal power for a variety of vectors ***θ***.

Searching for an optimal decision rule is a complex task because power depends on the *K*-dimensional ***θ***. It may be appropriate to focus on achieving high power under certain forms of ***θ***, particularly if ‘treatments’ are doses of a single compound. Rules attuned to situations where the treatment effects *θ*_1_,…,*θ*_*K*_ are high or low together may be thought of as ‘borrowing strength’ for inference about *θ*_*i*⋆_ from other stage 1 estimates 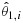, *i* ≠ *i*⋆. However, the correlations between 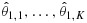, because of the common control arm in stage 1, also affect how these estimates should be weighted in the overall statistic 
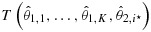
.

## 3 Methods for data combination

In this section, we outline the decision rules underlying six methods for data combination used in our numerical investigations of power. In our simulations, we have applied the futility stopping rule in (1) with *ℓ* = 0. We calibrated the critical values of all six decision rules so that tests attain overall type I error rate *α* when ***θ*** = (0,…,0) adjusting for the possibility of early stopping, arguing for each test that this ensures strong control of the FWER at level *α*. Therefore, the higher power achieved by a decision rule can be attributed to an efficient use of the available data rather than a higher type I error rate.

*Conventional test*: In the conventional approach with separate phase II and phase III studies, only phase III data are used in making the final decision to accept or reject 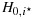. Let 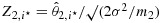 denote the standardized test statistic based on stage 2 data. To account for the possibility of stopping after stage 1 for futility, we reject 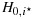
if

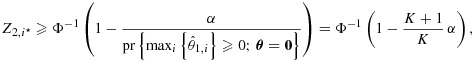
where Φ denotes the standard normal cumulative distribution function and **0** denotes the parameter vector (0,…,0). The overall type I error rate under ***θ*** = **0** is exactly *α*, and it follows from the arguments of Jennison and Turnbull 7, Section 3] that the FWER is controlled strongly at level *α*.
*TSE decision rule*: Adapting the procedure of Thall, Simon and Ellenberg 1 to a normal response, we define

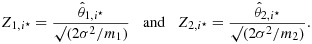
and reject 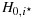
if

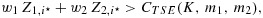
where *w*_*i*_=*√*{*m*_*i*_/(*m*_1_+*m*_2_)}, *i* = 1,2, and *C*_*TSE*_(*K*,*m*_1_,*m*_2_) is chosen to give FWER *α* when ***θ*** = **0**. Jennison and Turnbull 7 note that this ensures the FWER is controlled strongly at level *α*.
*Combination tests*: Bretz *et al*. 3 and Schmidli *et al*. 4 present a variety of adaptive designs for seamless phase II/III clinical trials. These proposals follow the general approach of Bauer and Kieser 8 for making mid-study data-dependent adaptations while preserving trial integrity. They use closed testing procedures 5 to control the FWER and combination tests 6 to conduct hypothesis tests using data from the two stages.
  Denote the set of indices *i* of null hypotheses *H*_0,*i*_ by 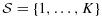. A closed testing procedure requires an *α*-level test of the intersection hypothesis 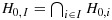 for each subset *I* of 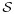; this test will reject *H*_0,*I*_ with probability at most *α* when all *H*_0,*i*_ with indices *i*∈*I* are true. Tests of intersection hypotheses *H*_0,*I*_ combine data from the two stages of the trial. A combination test of *H*_0,*I*_ is defined in terms of one-sided *p*-values *P*_1,*I*_ and *P*_2,*I*_ for *H*_0,*I*_ based on stage 1 and stage 2 data, respectively. Note that ‘stage 2 data’ refers to new data in stage 2, not the cumulative data at the end of stage 2. Using the inverse *χ*^2^ rule (originally proposed by Fisher 9 for combining separate experiments) to combine *p*-values in the test for each intersection hypothesis in the closed testing procedure, we obtain an overall decision rule that rejects 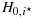 if

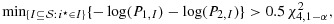
4
where 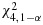 is the value exceeded with probability *α* by a 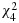 random variable. We refer to this as the ‘BK inverse *χ*^2^’ decision rule. Alternatively, using the inverse normal combination rule 10,11 to combine *p*-values gives the ‘BK inverse normal’ decision rule, which rejects 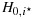 if

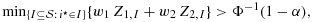
5
where, as in the TSE method, *w*_*i*_=*√*{*m*_*i*_/(*m*_1_+*m*_2_)}, *i* = 1,2. There are various choices for defining the *p*-values for intersection hypotheses in the previous methods. In our simulations, we compared the efficiencies of methods when Simes 12 and Dunnett 13 *p*-values are used for inter- section hypotheses.

Because the trial may stop for futility after stage 1, all the previous procedures have an FWER below *α*. Additional conservatism arises from using Simes' method to define *p*-values for intersection hypotheses arising from multiple comparisons with a common control 14. So as not to disadvantage methods using Simes' test in our investigations of decision rules, we have adjusted the critical values on the right-hand sides of (8) and (9) so that the FWER is *α* under ***θ*** = **0** proceeding on the assumption that this is a sufficient condition to ensure strong control of the FWER. In fact, it is difficult to give a general proof that the probability of a type I error is decreased when one treatment effect, say *θ*_*i*_, is increased above zero: because *H*_0,*i*_ is now false, rejecting it no longer counts as a type I error but, against this, a low *p*-value for treatment *i* may reduce the *p*-value for an intersection hypothesis involving a selected treatment *i*⋆≠*i*. We have used simulation to check the implications of these lower critical values in our examples, and in all cases, we found the type I error rate to be controlled at level *α* with some conservatism: see the supporting information accompanying this manuscript for further discussion.

We now illustrate the application of the previous testing procedures in an example.

## 4 Illustrative example

Liu and Pledger 15 discuss a seamless phase II/III trial comparing five doses of a treatment for migraine headaches against placebo. We simplify this example by assuming that both stages of the trial measure the same clinical endpoint, the decrease in monthly headache rate over 4 months. Responses are assumed to be normally distributed with standard deviation *σ* = 5. A reduction of 2 in the average monthly headache rate, compared with placebo, is taken to be clinically meaningful, and high power is desired to detect a dose with such an effect.

In our notation, *K* = 5 and for each dose *i* = 1,…,*K*, we wish to test *H*_0,*i*_: *θ*_*i*_≤0 against *θ*_*i*_>0. While controlling the FWER strongly at *α* = 0.025, we desire high power to select and declare efficacious a dose with a treatment effect *θ*_*i*_=2. Suppose the trial follows a two-stage design with *m*_1_=28 patients randomized to each dose and placebo in stage 1 and a further *m*_2_=140 allocated to each of dose *i*⋆ and placebo when sampling continues to stage 2. Such unequal division of resources between phases is common in practice, with larger sample sizes devoted to confirming efficacy of the selected dose in phase III.

Table I lists the critical values needed to implement the six decision rules described in section 3 with a familywise type I error rate of 0.025.

**Table I:** Critical values for six decision rules testing 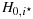: 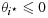 against 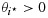.

Design	Test statistic	Critical value
Conventional	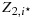	1.881
TSE	*w*_1_*Z*_1,*i*⋆_+*w*_2_*Z*_2,*i*⋆_	2.245
BK inverse *χ*^2^ (Simes)	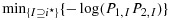	5.342
BK inverse *χ*^2^ (Dunnett)	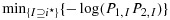	5.539
BK inverse normal (Simes)	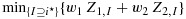	1.851
BK inverse normal (Dunnett)	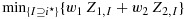	1.958

These rules control familywise type I error rate at level *α* = 0.025 for *K* = 5, *m*_1_=28, *m*_2_=140, *ℓ* = 0 and *σ* = 5.

**Figure 1:**
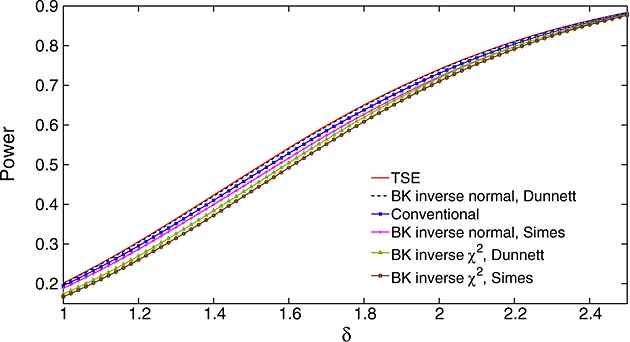
Power of six decision rules under *θ* = (0,0,0,0,*δ*) when *m*_1_=28, *m*_2_=140, *ℓ* = 0, *σ* = 5, and FWER is controlled strongly at *α* = 0.025. All estimates are based on 1 million simulations. The legend lists rules in order of decreasing power.

Suppose that only the highest dose gives an improvement over placebo and the vector of treatment effects has the form ***θ*** = (0,0,0,0,*δ*). Figure 1 shows the power of each decision rule, as a function of *δ*, to select dose 5 and reject *H*_0,5_. Results are based on 1 million replicates in each scenario considered, so standard errors of estimated probabilities are at most 0.0005. The TSE procedure is most powerful at all values of *δ*, closely followed by the BK inverse normal combination test using Dunnett *p*-values for intersection hypotheses. Surprisingly, the three other combination tests have lower power than the conventional test that does not use the phase II data at all. Differences in power are as high as 0.05 in places: the values of *δ* at which different rules attain the same power differ by up to 5% and, supposing the sample size needed to achieve a given power to be roughly proportional to *δ*^−2^, this translates into differences in sample size of up to 10%.

The results in Figure 1 parallel those of Jennison and Turnbull 16, Section 5.3] for an example with *K* = 4, *m*_1_=100, *m*_2_=500 and *σ* = 5 (although those authors did not consider methods using Dunnett tests). The failure in both examples of some decision rules to improve on the conventional test, which ignores stage 1 data, motivated our investigation of the underlying decision rules. We shall also investigate whether the same patterns of relative efficiency occur for other forms of ***θ*** and consider what is the optimal division of resources between phases II and III when the total sample size has been fixed. In order to explore these issues, we shall derive optimal decision rules for particular forms of ***θ*** and study the structure of these rules.

## 5 Optimal data combination rules

5.1. Optimizing power for a family of configurations of ***θ***

In the framework of section 2, the function 
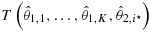
 in section 3 that this is a sufficient condition for some rules to provide strong control of the FWER but we shall have to check this property for the new rules that we derive.

We proceed by defining a Bayes decision problem with a prior distribution for ***θ*** and costs for each possible decision. We then search over values of these costs to find a version of this problem for which the optimal Bayes rule has type I error rate *α* under ***θ*** = **0** and so solves the problem originally stated in frequentist terms. The method of re-casting a frequentist problem as a Bayes decision problem has been used to find optimal group sequential tests; see, for example, 17–21. In our problem, power depends on a vector of treatment effects, and we handle this by dealing with a one-dimensional subset of vectors ***θ*** at a time. This provides a benchmark for each family of ***θ*** vectors, against which other decision rules can be compared. While it is desirable to have a single rule with robust efficiency in a wide variety of situations, it could be that quite different rules are needed to achieve high power for different configurations of ***θ***, in which case, the importance of these different scenarios should guide the overall choice.

For our first problem, with ***θ*** a permutation of (*γδ*,…,*γδ*,*δ*), let ***ξ***_*i*_ denote the vector with *θ*_*i*_=*δ* and the other *K* − 1 elements equal to *γδ*. Define a prior distribution for ***θ*** with discrete mass function *π*(***θ***) placing probability 1/(*K* + 1) on each of the cases ***θ*** = **0** and ***θ*** = ***ξ***_*i*_, *i* = 1,…,*K*. The only hypothesis that can be rejected when treatment *i* is selected for stage 2 is *H*_0,*i*_. Thus, the set of possible actions is {*A*_0_,*A*_1_,…,*A*_*K*_} where, for 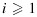, *A*_*i*_ means that treatment *i* is selected after stage 1 and *H*_0,*i*_ is rejected at the end of stage 2, while *A*_0_ indicates stopping for futility at stage 1 or continuing to stage 2 but failing to reject any *H*_0,*i*_. We define the loss function *L*(***θ***,*A*) as

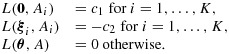

The reward for correctly rejecting *H*_0,*i*_ appears as the negative cost −*c*_2_, and the penalty for failing to reject *H*_0,*i*_ when ***θ*** = ***ξ***_*i*_ is the absence of this reward. Our original criteria concern power to declare efficacy of treatment *i* when ***θ*** = ***ξ***_*i*_ but do not differentiate between ways of failing to reject *H*_0,*i*_ when ***θ*** = ***ξ***_*i*_; hence, we define the same loss, of zero, for actions *A*_0_ and *A*_*j*_, 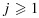 and *j* ≠ *i*, in this case.

The Bayes rule for the problem that we have defined minimizes the Bayes risk

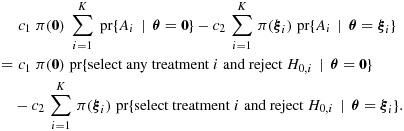
6
Suppose that treatment *i*⋆ is selected and data at the end of stage 2 are summarized as

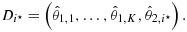

Either action *A*_0_ or action 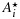 must be taken. Let 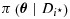 denote the posterior distribution of ***θ*** given data 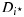. If action 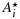 is chosen, so 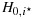 is rejected, the posterior expected loss is

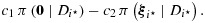
7

All costs associated with action *A*_0_ are zero, so if this action is chosen and 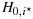 is not rejected, the loss is exactly zero. Thus, the Bayes rule that minimizes (11) rejects 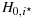 if and only if (13) is negative or, equivalently, if

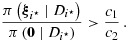
8
Because the prior probabilities of ***θ*** = **0** and ***θ*** = ***ξ***_*i*⋆_ are equal, the left-hand side of (14) is simply the likelihood ratio of the observed data under ***θ*** = ***ξ***_*i*⋆_ and ***θ*** = **0**.

Given ***θ***, the stage 1 estimates 
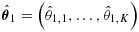
 are distributed as 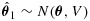, where *V*_*ii*_=(2*σ*^2^/*m*_1_) for *i* = 1,…,*K* and 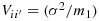 for *i* ≠ *i*^′^. The inverse of *V* has elements

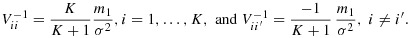

The log likelihood ratio of 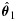 under ***θ*** = ***ξ***_*i*⋆_ and ***θ*** = **0** is

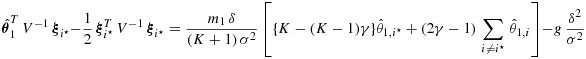
9
for some constant *g*. The log likelihood ratio for the stage 2 data 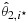 is

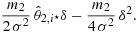
10
Adding (16) and (17) gives the log likelihood ratio of *D*_*i*⋆_ under ***θ*** = ***ξ***_*i*⋆_ and ***θ*** = **0**. It follows that the condition for the Bayes test to reject *H*_0,*i*⋆_ can be written as

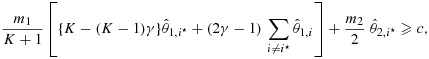
11
where

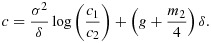
12
The constant *c* is an increasing function of the ratio *c*_1_/*c*_2_. Also, *c* depends on *δ*, but the expression on the left-hand side of (18) does not.

Suppose *c* is such that the rule given by (18) has type I error rate *α* when ***θ*** = **0**. For any given *δ*, there are costs *c*_1_ and *c*_2_ that satisfy (19) with this *c*. Hence, the decision rule (18) minimizes (11) for this *δ* and the pair (*c*_1_,*c*_2_), and so, it maximizes

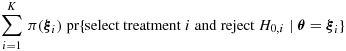

amongst all rules with type I error rate less than or equal to *α* when ***θ*** = **0**. Thus, this decision rule solves the problem posed at the start of this section, and we note that, by construction, the same rule is optimal for all values of *δ*.

We can find this optimal rule by searching for the constant *c* in section 4 where ***θ*** is a permutation of (0,…,0,*δ*). In this case, estimates 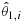 for treatments other than *i*⋆ have negative weights in section 3, strong control of the FWER does follow from controlling the type I error rate at ***θ*** = **0** in this case. When 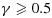, the weight of each 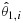 is positive and, by the arguments applied for Simes' rule in section 3, we expect that controlling the type I error rate at ***θ*** = **0** implies strong control of the FWER for all possible vectors ***θ***.

**Figure 2:**
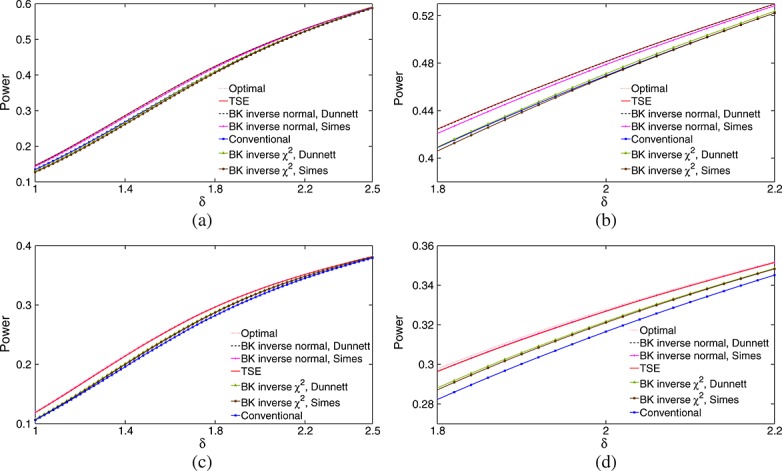
Power achieved by decision rules in the example of section 4 when *θ* is a random permutation of (*γ*,*γ*,*γ*,*γ*,1)*δ* with (a) - (b) *γ* = 0.5 and (c) - (d) *γ* = 0.75. Decision rules are listed in order of decreasing power. Results are based on 1 million simulations for each scenario.

Figure 2 compares power curves of optimal decision rules and the six methods introduced in section 4, with *K* = 5 treatments and a control. Panels (a) and (b) of Figure 2 show power curves for *γ* = 0.5, the case in which the TSE rule is optimal.

We see that the two BK inverse normal combination tests have almost the same power as the TSE rule: for the test using Simes' rule, this is a significant improvement over the case *γ* = 0 seen in Figure 1. However, the two BK inverse *χ*^2^ combination tests still have lower power than the conventional test using stage 2 data only. The power curves for *γ* = 0.75 in panels (c) and (d) show the TSE rule and the two BK inverse normal combination tests to be almost as powerful as the optimal decision rule for this case, and now the two BK inverse *χ*^2^ combination tests have a small advantage over the conventional test.

The power curves for *γ* = 0.75 are noticeably lower than for *γ* = 0.5 because of the higher probability of a sub-optimal treatment being selected after stage 1. If a final decision in favour of a sub-optimal treatment with sufficiently high effect size is deemed acceptable, the definition of power could be modified to include this. Such a definition would certainly be reasonable in the limit as *γ*→1.

5.2 Optimizing power for general configurations of ***θ***

The approach of Section 5.1 can be extended to obtain decision rules that maximize power averaged over the *K*! permutations of (*γ*_1_,…,*γ*_*K* − 1_,1)*δ*, where 0≤*γ*_1_<…<*γ*_*K* − 1_<1, subject to a type I error rate of at most *α* when ***θ*** = **0**. As before, power is defined to be the probability of selecting the treatment *i*_max_ with the highest effect and then rejecting 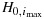. The optimal decision rule can then be examined to check whether controlling the type I error at ***θ*** = **0** implies strong control of the FWER.

Let *Q* denote the set of *K*! parameter vectors ***θ*** obtained by permuting the elements of (*γ*_1_,…,*γ*_*K* − 1_,1)*δ*. In our Bayes decision problem, we define the prior distribution *π*(***θ***) on ***θ***∈*Q* to give probability 1/(*K* + 1) to ***θ*** = **0** and 1/{(*K* + 1)(*K* − 1)!} to each element of *Q*. For *i* = 1,…,*K*, let *Q*_*i*_ be the subset of *Q* containing vectors ***θ*** with *θ*_*i*_=*δ*. With actions *A*_0_,*A*_1_,…,*A*_*K*_ as defined in Section 5.1, we define the loss function *L*(***θ***,*A*) to be

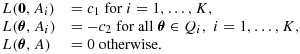

When treatment *i*⋆ is selected in stage 1, either action *A*_0_ or 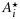 must be taken after stage 2. We seek the Bayes rule that minimizes the Bayes risk

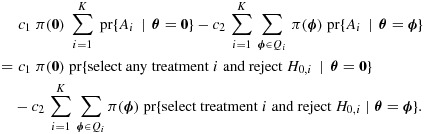

If *c*_1_ and *c*_2_ are chosen so that the Bayes optimal rule has type I error rate *α* when ***θ*** = **0**, we can deduce that this rule maximizes

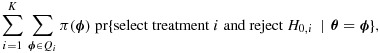

and it therefore maximizes the average power over the *K*! permutations of (*γ*_1_,…,*γ*_*K* − 1_,1)*δ*, amongst all decision rules with type I error rate at most *α* at ***θ*** = **0**.

As before, taking action *A*_0_ after stage 2 has cost zero. The posterior expected loss for action *A*_*i*⋆_ given that treatment *i*⋆ is selected and data *D*_*i*⋆_ are observed is

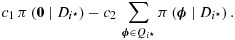

It follows that the Bayes rule rejects *H*_0,*i*⋆_ if

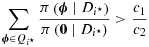

or, equivalently, if

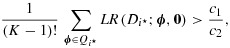
13
where 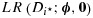 denotes the likelihood ratio of data *D*_*i*⋆_ under parameter vectors ***θ*** = ***φ*** and ***θ*** = **0**.

Given ***θ***, the stage 1 estimates 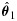 follow an *N*(***θ***,*V*) distribution. The inverse of *V* can be written as

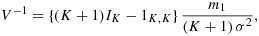

where *I*_*K*_ is the *K* × *K* identity matrix and 1_*K*,*K*_ the *K* × *K* matrix with all elements equal to 1. Thus, the log likelihood ratio of the stage 1 data under ***θ*** = ***φ*** and **0** is

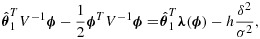
14
where

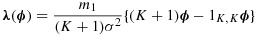
15
and the constant *h* is the same for all vectors ***φ***∈*Q*.

When ***φ***∈*Q*_*i*⋆_, and so *φ*_*i*⋆_=*δ*, the log likelihood ratio for the stage 2 data under ***θ*** = ***φ*** and ***θ*** = **0** is

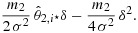
16
Combining (28) and (30), the condition (26) can be written as

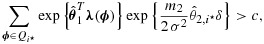
17
where *c* depends on *δ*. The value of *c* for which the type I error rate is *α* under ***θ*** = **0** varies with *δ*. Therefore, no uniformly most powerful decision rule exists for the ***θ*** configuration, and we find the appropriate critical value at each *δ* value of interest using the Robbins–Monro algorithm. Although the left-hand side of (31) involves a sum of (*K* − 1)! terms, this poses no real computational difficulty for typical values of *K*.

In order for an optimal decision rule to protect the FWER over the whole parameter space, coefficients of all elements of 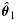 must be non-negative in each term 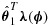. Because the smallest coefficient is *m*_1_/{(*K* + 1)*σ*^2^} times

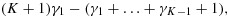
18
we simply require that the expression section 3 to claim that a rule of the form section 4.

**Figure 3:**
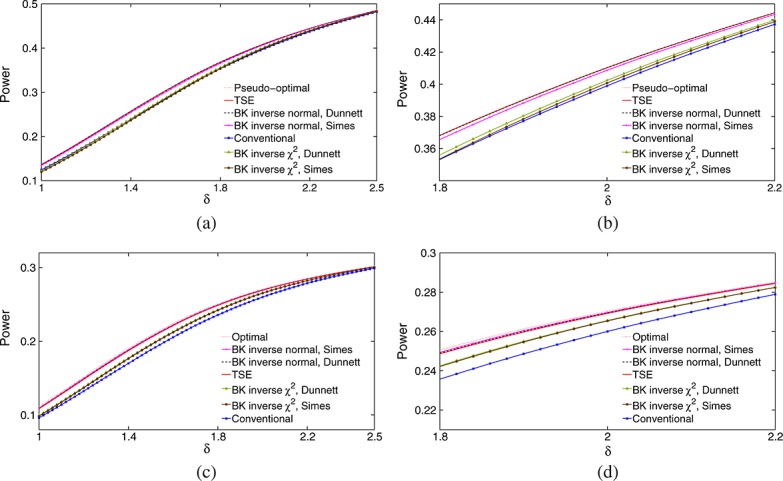
Power achieved by decision rules in the example of section 4 when *θ* is a random permutation (a) - (b) of (0.3,0.475,0.65,0.825,1)*δ* and (c) - (d) of (0.75,0.8125,0.875,0.9375,1)*δ*. Decision rules are listed in order of decreasing power. Results are based on 1 million simulations for each scenario.

In panels (a) and (b), the *γ*_*j*_s are equally spaced between 0.3 and 1. As the form of the optimal rule varies with *δ*, each point on the power curve evaluates the rule maximizing power at that particular value of *δ*. As some 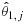
s have negative weights in (31), these rules do not provide strong control of the FWER and we label them as ‘pseudo-optimal’. The curve sets an upper bound for the power that can be attained and we deduce that the TSE rule and the two BK inverse normal combination tests have close to the maximum possible power. Indeed, the performance of these three procedures is impressive in view of the fact that they do not have the flexibility of the ‘pseudo-optimal’ rules to adapt to *δ*. In panels (c) and (d), where the *γ*_*j*_s are equally spaced between 0.75 and 1, *γ*_1_>*K*/(*K* + 2) so the rules given by (31) attach positive weights to all *θ*_*j*_s and we take them to be truly optimal. Again, the TSE rule and the two BK inverse normal combination tests have close to maximum power. The efficiency of the inverse normal combination test using Simes' rule in these examples indicates that the treatment effects of sub-optimal treatments are now sufficiently high that it is beneficial for the final decision rule to ‘borrow strength’ from 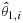, *i* ≠ *i*⋆.

### 5.3 Optimizing power under dose–response assumptions

We now consider the situation where investigators suspect that a particular pattern of treatment effects may occur but these views are not held strongly enough to change the form of the study design from that described in section 2. We shall consider the case where the treatment effect is expected to increase steadily with dose but side-effects or poorer compliance at higher doses may disrupt this relationship. It is of interest to know whether using such information about the likely pattern of treatment effects can lead to a significant increase in power.

We capture this somewhat equivocal view about possible treatment effects by formulating a Bayes decision problem with a special prior distribution. Let 0≤*γ*_1_<…<*γ*_*K* − 1_<1 be specified and suppose the parameter vector ***θ*** is either **0** or a permutation of (*γ*_1_,…,*γ*_*K* − 1_,1)*δ*. We assign prior probability 1/(*K* + 1) to ***θ*** = **0** and allocate probability 1/(*K* + 1) to each of the cases *θ*_*i*_=*δ*, *i* = 1,…,*K*. For *i* = *K*, the maximum treatment effect is *θ*_*K*_=*δ*, and we assign all the probability 1/(*K* + 1) to the case ***θ*** = (*γ*_1_,…,*γ*_*K* − 1_,1)*δ* = ***θ***_*ord*_, say, in line with the assumption that treatment effects increase with dose. For *i* < *K*, the maximum effect is not at the maximum dose, and, because the pattern cannot be monotone, we divide the prior probability 1/(*K* + 1) evenly across the (*K* − 1)! permutations of (*γ*_1_,…,*γ*_*K* − 1_,1)*δ* with *θ*_*i*_=*δ*. Thus, the difference between this prior and that used in Section 5.2 is that the probabilities 1/{(*K* + 1)(*K* − 1)!} for vectors ***θ*** in the subset *Q*_*K*_ are re-allocated to the single vector ***θ***_*ord*_, capturing the desired knowledge about the order of treatment effects in this case.

We define the same loss function as in Section 5.2 and find the Bayes optimal decision rule. The choice of prior implies that the Bayes rule rejects *H*_0,*i*⋆_ if

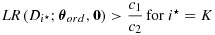

and if

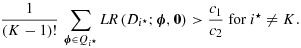

As in Section 5.2, the likelihood ratio for data *D*_*i*⋆_ can be written as

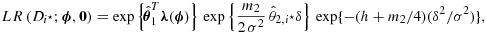

where ***λ***(***φ***) is as defined in (29) and this is used with vectors ***φ***∈*Q*_*i*⋆_ for *i*⋆≠*K* and with ***φ*** = ***θ***_*ord*_ for *i*⋆=*K*.

The form of the Bayes rule depends on *δ* so that no uniformly most powerful test exists for treatment effect configuration ***θ***_*ord*_. At each positive value of *δ*, the Robbins–Monro algorithm can be used to find the appropriate choice of *c*_1_/*c*_2_ that gives an optimal decision rule with type I error rate *α* at ***θ*** = **0**. In order for optimal decision rules to protect the FWER over the whole parameter space, coefficients of elements of 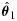 must be non-negative in each term 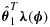 for ***φ***∈*Q*_*i*⋆_ or ***φ*** = ***θ***_*ord*_, so *γ*_1_,…,*γ*_*K*_ must satisfy the same conditions discussed in Section 5.2.

We have calculated power curves for decision rules of the previous form when ***θ*** = ***θ***_*ord*_ and the values of *γ*_1_,…,*γ*_*K* − 1_ are as in cases (a) and (c) of Figure 3. When the correct treatment, *i*⋆=*K*, is selected, the decision depends on 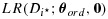 and so takes full advantage of the monotonicity assumption. We compared power under ***θ*** = ***θ***_*ord*_ with that of the optimum rules with no monotonicity assumption, derived in Section 5.2. In case (a), where effect sizes range from 0.3*δ* to *δ*, the maximum increase in power from use of dose–response information is 0.005; although the conditions for strong control of the FWER over the whole parameter space are not met, this is the case for both types of procedure so comparisons are fair. In case (c), effect sizes are closer, all tests control the FWER strongly, and the maximum increase in power is much smaller at 0.0005 (coupling of simulations of the different methods implies that this difference is still estimated reliably). The other six methods are unaffected by assumptions about the possible monotonicity of ***θ***. However, because these assumptions lead to such small improvements, power curves for the new optimal rules are barely distinguishable from those shown in Figure 3 for the pseudo-optimal rules in (a) and the optimal rules in (c), and the TSE rule and both inverse normal rules remain very close to optimal.

Bretz *et al*. 23 propose methods that accommodate uncertain information about a dose–response curve by assuming that this curve belongs to a specified set of models 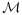. In their multiple comparison procedures with modelling techniques (MCP-Mod) approach, they define a test statistic *T*_*m*_ appropriate to each model 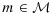 and use max*mT*_*m*_ as a global statistic to test for a positive dose–response relationship. The adjusted *p*-value is calculated using the joint distribution of the *T*_*m*_, 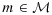, when the treatment effect is zero at all doses, that is, ***θ*** = **0** in our notation. Because the *T*_*m*_ are weighted sums of mean responses at each dose and some means can have negative coefficients, the FWER is not controlled strongly for all treatment effect vectors ***θ***. Assuming that all treatment effects have the same sign resolves this problem: the same assumption would justify use of the ‘pseudo-optimal’ tests in case (a).

Our results show that robustly efficient methods such as the TSE rule achieve most of the potential gains from additional dose–response assumptions: the parallel in the setting of Bretz *et al*. 23 would be to take the maximum observed effect over all doses as the global test statistic. Bretz *et al*. found their method to have comparable power to a certain likelihood ratio test in many cases. Their method has a noticeable advantage when the effect size decreases at high doses, which is to be expected as the likelihood ratio test relies on a monotonicity assumption: the TSE rule makes no such assumption and should not be misled in such cases.

We acknowledge that our setting differs from that of Bretz *et al*. 23 in having two stages, and the gains from model information in stage 1 become diluted in the overall power. Also, Bretz *et al*. 23 made further use of their modelling framework by identifying the model producing the maximum *T*_*m*_ and using this model to select the minimum dose achieving a certain specified effect size for further testing. We shall return to discussion of such objectives in Section 9.

## 6 Relative efficiencies of data combination rules

We can express the power differences between decision rules in terms of the sample size needed to achieve a specific power. With the design of section 2 and group sizes *m*_1_ and *m*_2_, we have derived optimal decision rules under particular assumptions about the vector of treatment effects, ***θ***. Suppose the optimal rule achieves power 1 − *β* for a given form of ***θ*** with maximum treatment effect *δ*. If another rule requires group sizes to be increased to *ρm*_1_ and *ρm*_2_ in order to achieve the same power, the relative efficiency of this rule, expressed as a percentage, is 100/*ρ*.

We have calculated the efficiency of decision rules applied to the example of section 4 where *K* = 5, *α* = 0.025, *m*_1_=28 and *m*_2_=140. Table II lists relative efficiencies of the six decision rules of section 3 for eight configurations of ***θ*** in which the highest treatment effect is *δ* = 2 (fixing power at a different value of *δ* has only a small effect on our conclusions).

**Table II:** Efficiencies of six decision rules when the treatment vector is a permutation of (a) 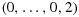, (b) 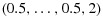, (c) 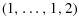, (d) 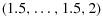, (e) (0.6,0.95,1.3,1.65,2), (f) (1.5,1.625,1.75,1.875,2), (g) (0.6,0.95,1.3,1.65,2) and (h) (1.5,1.625,1.75,1.875,2).

	Treatment effect vector							
Combination rule	a	b	c	d	e^1^	f	g^1^	h
TSE	100	100	100	99	100	98	99	98
BK inverse normal, Dunnett	100	100	100	99	100	99	99	99
BK inverse normal, Simes	95	98	99	99	99	99	98	99
BK inverse *χ*^2^, Dunnett	95	96	96	96	96	95	95	95
BK inverse *χ*^2^, Simes	93	95	96	96	95	95	95	95
Conventional test	97	97	96	93	94	90	94	90

In cases (g) and (h), optimal decision rules use information about the order of the elements of ***θ***. Group sizes are *m*_1_=28 and *m*_2_=140. Results are based on 1 million simulations.

1 Comparisons in (e) and (g) are with pseudo-optimal decision rules, which do not provide strong control of the familywise error rate, so entries are lower bounds on actual efficiencies.

Cases (a) to (d) are for ***θ*** of the form considered in Section 5.1. The TSE rule is optimal for case (c), and, in line with the conjecture made in Section 5.1, we also treat it as being optimal for cases (a) and (b). The form of ***θ*** in cases (e) and (f) is that considered in Section 5.2. In case (e), we calculated efficiency relative to the ‘pseudo-optimal’ decision rule. This rule does not control the FWER strongly for all ***θ***, but it provides an upper bound on the attainable power, and this is a rather tight upper bound as two rules that do protect FWER have close to 100% efficiency. Cases (g) and (h) concern the situation of section 3 where there is partial information about the order of treatment effects; in (g), we are only able to derive a ‘pseudo-optimal’ decision rule, and we report efficiency relative to the upper bound this rule provides.

We have carried out the same efficiency assessments with group sizes *m*_1_=56 and *m*_2_=112, and the parallel results are presented in Table III.

**Table III:** Efficiencies of six decision rules when the treatment vector is a permutation of (a) 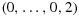, (b) 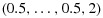, (c) 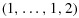, (d) 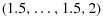, (e) (0.6,0.95,1.3,1.65,2), (f) (1.5,1.625,1.75,1.875,2), (g) (0.6,0.95,1.3,1.65,2) and (h) (1.5,1.625,1.75,1.875,2).

	Treatment effect vector							
Combination rule	a	b	c	d	e^1^	f	g^1^	h
TSE	100	100	100	99	99	98	98	97
BK inverse normal, Dunnett	99	99	100	99	99	98	98	98
BK inverse normal, Simes	91	95	98	99	98	99	97	98
BK inverse *χ*^2^, Dunnett	96	97	97	97	97	96	95	96
BK inverse *χ*^2^, Simes	93	95	97	97	96	96	95	96
Conventional test	89	89	89	85	86	82	85	82

In cases (g) and (h), optimal decision rules use information about the order of the elements of ***θ***. Group sizes are *m*_1_=56 and *m*_2_=112. Results are based on 1 million simulations.

1 Comparisons in (e) and (g) are with pseudo-optimal decision rules, which do not provide strong control of the familywise error rate, so entries are lower bounds on actual efficiencies.

Here, the conventional procedure is less efficient, which is to be expected because more patients are treated in stage 1 and there is greater potential benefit in using their data in the final analysis. The inverse *χ*^2^ tests, which give equal weight to stage 1 and stage 2 data summaries, fare better with these values of *m*_1_ and *m*_2_. However, in all cases where a Dunnett test is used for intersection hypotheses, the inverse*χ*^2^ combination test still lags behind the inverse normal rule. There is just one example (case (a) of Table III) of a Simes test, for which the inverse *χ*^2^ combination test is superior to the inverse normal, but then the Dunnett test with an inverse normal combination rule is far superior.

Results in Tables  II and  III show that use of stage 1 data in the final analysis can lead to worthwhile gains in efficiency over the conventional test based on stage 2 data alone. The methods of choice are the TSE procedure and the BK inverse normal combination test using Dunnett tests for intersection hypotheses: these decision rules attain close to the maximum possible power in all scenarios, with relative efficiency of at least 97% in all cases and 99% or more in the majority of cases. The BK inverse normal method using Simes' tests for intersection hypotheses performs poorly when there is a single treatment with a high effect size, but this rule can be close to optimal in other situations. We do not recommend decision rules based on inverse *χ*^2^ combination tests: in all but extreme cases, these are dominated by the rules using inverse normal combination tests (using Dunnett or Simes *p*-values for intersection hypotheses) and, in some situations, gain no advantage at all from the use of stage 1 data.

## 7 Optimal division of sample size between phases II and III

**Figure 4:**
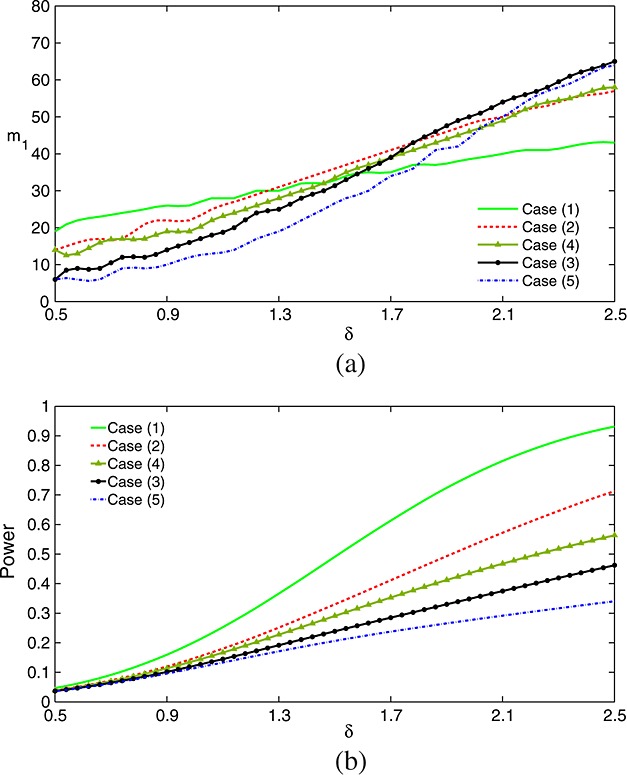
(a) Stage 1 group sizes maximizing the power of the TSE procedure when the total sample size is fixed at 448 and *θ* is a random permutation (1) of (0,0,0,0,1)*δ*, (2) of (0.5,0.5,0.5,0.5,1)*δ*, (3) of (0.75,0.75,0.75,0.75,1)*δ*, (4) of (0.3,0.475,0.65,0.825,1)*δ* and (5) of (0.75,0.8125,0.875,0.9375,1)*δ*. (b) Power achieved by the optimized TSE procedures. Decision rules are listed in order of decreasing power. Designs are specified with *K* = 5, *ℓ* = 0, *σ* = 5.0 and *α* = 0.025. Results are based on 1 million simulations for each scenario.

Suppose that in the previous example *K* = 5 and *α* = 0.025 are fixed but the group sizes *m*_1_ and *m*_2_ can be chosen freely subject to an upper bound on the total sample size (*K* + 1)*m*_1_+2*m*_2_. We shall restrict attention here to the robustly efficient TSE procedure. For this decision rule, we have calculated values of *m*_1_ and *m*_2_ that optimize power when the total sample size is fixed at 448, as in the example of section 4. Figure 4 plots the value of *m*_1_ that maximizes power, as defined in (4), for a variety of treatment means ***θ***.

Optimal values of *m*_1_ were found by a direct search over the integers between 1 and 74; the accuracy of comparisons was enhanced by using the same sequence of pseudo-random numbers to simulate the power of each design. Thall, Simon and Ellenberg 1 report design settings that minimize the expected sample size of the TSE procedure when ***θ*** has the form (*γ*_1_,…,*γ*_1_,1)*δ*. In Figure 4, we present results for a wider variety of configurations for ***θ***. Given the robust efficiency of the TSE procedure, we expect these values of *m*_1_ will also be close to optimal for the optimal tests of Section 5 and for the inverse normal combination rule with Dunnett *p*-values.

**Figure 5:**
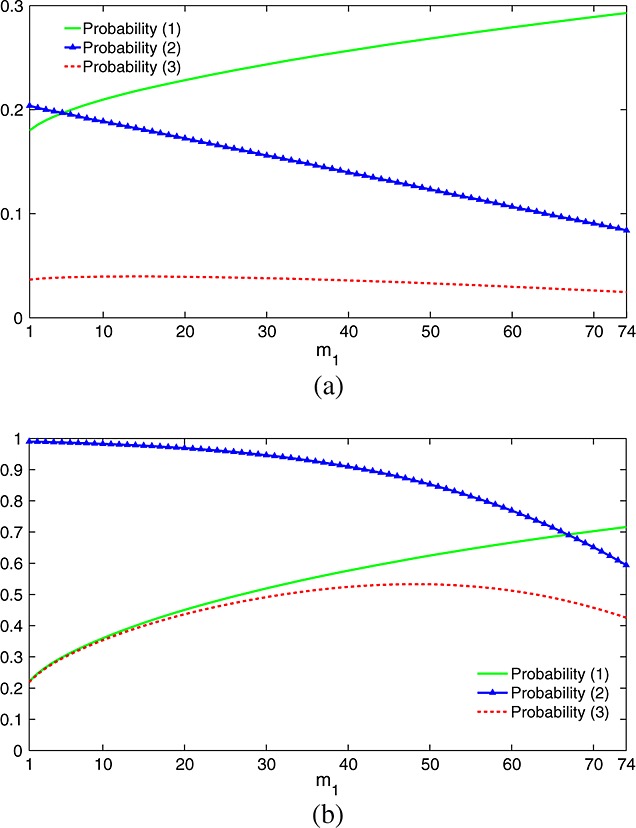
Operating characteristics of the TSE procedure with fixed total sample size *N* = 448 when *θ* = (0.5,…,0.5,1)*δ* with (a) *δ* = 0.5 and (b) *δ* = 2.0. Plotted probabilities are as follows: (1) pr{select treatment *K*;*θ*}, (2) pr{reject *H*_0,*K*_|treatment *K* selected;*θ*} and (3) the product of these, namely pr{select treatment *K* and reject *H*_0,*K*_;*θ*}. Designs have *K* = 5, *ℓ* = 0, *σ* = 5.0 and *α* = 0.025. Results are based on 1 million simulations for each scenario.

The optimum *m*_1_ varies with both the shape of the vector ***θ*** and the scale factor *δ*. When selecting *m*_1_, we trade accuracy in selecting the best treatment, *i*_*max*_, in stage 1 with sample size for comparing this treatment against control in stage 2. Figure 5 illustrates the consequences of this trade-off when ***θ*** = (0.5,…,0.5,1)*δ* with *δ* = 0.5 and *δ* = 2.0.

Initially, power increases with *m*_1_ because of the increased selection accuracy. However, increasing *m*_1_ also reduces the total number of observations on treatment *i*_*max*_ when this treatment is selected, and this eventually results in a loss of overall power to reject 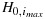. The same considerations help explain why the optimum *m*_1_ increases with *δ*: when *δ* is large, modest values of *m*_2_ still give a high conditional probability of rejecting 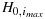 when treatment *i*_*max*_ is selected, thus we can take a larger value of *m*_1_ to improve the probability of selecting treatment *i*_*max*_ in stage 1. In the example, the optimum *m*_1_ for *δ* = 0.5 is 14, while that for *δ* = 2.0 is 49.

Because optimum values of *m*_1_ can be below 10 or above 60, we conclude that no single choice is close to ideal in all scenarios. Rather, investigators should consider the most likely scenarios, not necessarily of the form (*γ*,…,*γ*,1)*δ*, for their trial and choose group sizes that will give the best average power across these cases. Such scenarios could be established by conducting a Bayesian prior elicitation meeting ahead of the phase II/III trial to explore experts' prior opinion on the efficacy of the *K* treatments relative to control. With a given decision rule, it is straightforward to run simulations to compare different choices of *m*_1_ and choose a value that will provide good power under an anticipated set of treatment effects. Note that our definition of power gives no reward for selecting a good second-best treatment and rejecting its null hypothesis although, in practice, this might be considered a successful outcome. This is not a major issue for most of the configurations of ***θ*** described in Figure 4, where treatment *i*_*max*_ is superior to its nearest competitor by some margin. However, when considering cases where several treatments are competitive, it may be appropriate to use an alternative definition of power and, for example, choose group sizes to maximize the probability of selecting any treatment *i* with a treatment effect within 10% of the largest treatment effect and then rejecting *H*_0,*i*_.

## 8 Value of phase II data in the final analysis

The relative efficiencies in Tables II and III are based on comparing trial designs where both stage 1 and stage 2 group sizes, *m*_1_ and *m*_2_, are multiplied by a common factor. Another way to assess the benefits of a seamless phase II/III design is to determine how many additional phase III observations would be needed to achieve the increase in power gained by using phase II data in the final analysis. We shall make this assessment when the TSE decision rule is used.

For a given vector of treatment effects ***θ***, we can calculate the stage 2 sample size 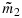 such that selecting a treatment based on *m*_1_ stage 1 observations and then applying a conventional test with 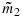 stage 2 observations on the selected treatment and control gives the same overall power as the TSE rule with group sizes, *m*_1_ and *m*_2_. Thus, the 2*m*_1_ stage 1 observations on treatment *i*⋆ and control in the TSE decision rule have the same benefit as an additional 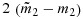 stage 2 observations for the conventional test. We express the percentage value of the stage 1 observations on treatment *i*⋆ and control relative to extra stage 2 observations in a conventional design as

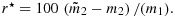

**Figure 6:**
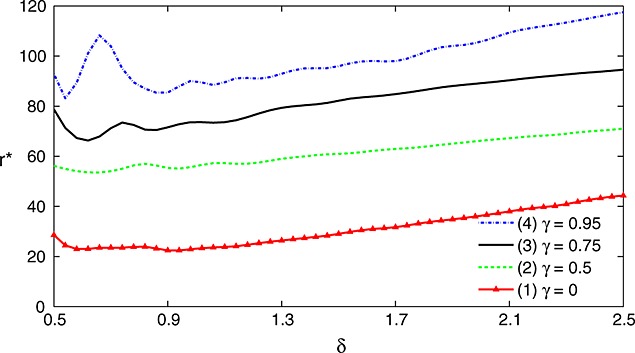
Percentage value, *r*⋆, of stage 1 data used in the TSE rule, relative to additional stage 2 observations for *θ* = (*γ*,…,*γ*,1)*δ* with (1) *γ* = 0, (2) *γ* = 0.5, (3) *γ* = 0.75 and (4) *γ* = 0.95. Designs are as specified in section 2 with *K* = 5, fixed total sample size *N* = 448, (*m*_1_,*m*_2_) chosen to maximize the power of the TSE procedure, *ℓ* = 0, *σ* = 5.0 and *α* = 0.025. Results are based on 1 million simulations for each scenario.

Figure 6 shows plots of *r*⋆ against *δ* for the example of Section 4 when ***θ*** is of the form (*γ*,…,*γ*,1)*δ* and, for each value of *δ*, *m*_1_ and *m*_2_ are chosen to maximize the power of the TSE procedure subject to a fixed total sample size *N* = 448.

Results vary with the form of ***θ*** and values of *r*⋆ at *δ* = 1 rise from 22 when *γ* = 0 to almost 100 when *γ* = 0.95. The critical value in the TSE decision rule, calculated under ***θ*** = (0,…,0), adjusts for multiple testing and so avoids any bias from selecting the treatment with the best stage 1 results. When *γ* = 0, the treatment with effect size *δ* is very likely to be chosen and the adjustment for multiple testing reduces power, leading to a low *r*⋆. For higher values of *γ*, the treatment with effect size *δ* must outperform its rivals in order to be selected after stage 1: it is then likely to have an above average estimate, 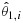, and this balances the effect of the multiplicity adjustment. As *γ*→1, *r*⋆ can exceed 100, indicating that information from all *K* treatments, not just treatment *i*⋆ and the control, contributes to the final decision.

In a trial involving multiple treatments or several doses of a single treatment, one might expect the treatment effects to be spread out between zero and the highest value. Thus, of the scenarios in Figure 6, case (2), with *γ* = 0.5, represents the most plausible situation. In this case, the stage 1 responses on treatment *i*⋆ have an equivalent value to around 60% of their number of stage 2 observations. Recognizing the usual uncertainty about likely treatment effects, we suggest that stage 1 data on treatment *i*⋆ should typically be viewed as offering around 50% to 70% of their face value as stage 2 observations in many situations. Since the TSE rule is close to optimal under general configurations of ***θ***, the results of this section should also provide an accurate reflection of the value of using stage 1 data in other efficient decision rules.

## 9 Discussion

We have sought optimal data combination rules for seamless two-stage designs, making the problem tractable by focusing on one configuration of ***θ*** at a time. In many situations, the optimal rules that we have derived control the FWER strongly; in other cases, our results provide upper bounds on the attainable power that serve as benchmarks for other procedures. We have identified two decision rules that we would recommend for use in practice, namely the TSE procedure and the BK inverse normal combination test using Dunnett *p*-values for intersection hypotheses. These rules are highly efficient in a variety of situations and tailoring the decision rule to the particular configuration of ***θ*** can give only very small additional efficiency. Furthermore, because both rules can be expressed as closed testing procedures, they can be used flexibly, still controlling the FWER when additional criteria are used to select a treatment for stage 2. We have also demonstrated how observations can be divided between the two stages to maximize power in a given scenario. Comparisons with the conventional practice of using phase III data alone in a final hypothesis test confirm that combining data across phases can improve power: for typical vectors of treatment effects, the increase in power is comparable with that achieved by adding 50% to 70% of the subjects on two stage 1 treatments to the stage 2 sample size and performing a conventional analysis. We have reached similar conclusions in simulations with different numbers of treatments and different sample sizes. In some situations, the benefits of data combination may be deemed insufficient to compensate for the planning and logistical effort involved in a seamless phase II/III trial; in others, particularly clinical trials for rare diseases, the power gained from stage 1 data may be deemed very worthwhile.

Sampson and Sill 2 derived a conditionally unbiased most powerful test for this problem. Their conditioning event is rather complex, as is the resulting test, and their procedure does not include stopping for futility after stage 1. If adapted to our problem, this method could not do better than our optimal rules for particular ***θ*** vectors. Bretz *et al*. 24 asked whether the approach of Sampson and Sill 2 could be extended to find an unconditionally unbiased most powerful test: our results show that different tests are optimal for different configurations of treatment effects, so this is not the case.

Extensions of the problem described in section 2 have been proposed and studied. Optimizing procedures in more complex settings may not be feasible, but, to the extent that these new problems retain core elements of the basic problem, we expect our conclusions to remain relevant. As an example, Bischoff and Miller 25 consider the case of two treatments and a control with normal responses of unknown variance, and they tailor the design to minimize total expected sample size. Their test statistic combines estimates of the effect of the selected treatment from stages 1 and 2 in the same way as the TSE rule, so our results suggest that using this estimate in a *t*-statistic will give good power.

Stallard and Todd 26 consider testing multiple treatments against a control in a sequential design in which the most promising treatment is selected at the first analysis and subsequent interim analyses allow early stopping for a final decision. Calculations follow similar lines to those of standard group sequential tests; see, for example, 27, Ch. 19]. With just two analyses, this method reduces to the TSE procedure, and we conclude that it combines data before and after treatment selection in an efficient way. The approach can be extended in various directions: these include allowing treatments to be dropped over several analyses 28,29 or basing the treatment selection on a short-term endpoint 30.

Magirr *et al*. 31 propose a new type of trial design for comparing multiple treatments with a control at multiple analyses. As in the TSE procedure, decision rules are defined in terms of the means of cumulative data on each treatment and the control. An innovative approach to computation makes it feasible to create designs comparing many treatments with several interim analyses. Wason and Jaki 32 use numerical search methods to optimize features of these designs, including the allocation ratio between active treatments and the control.

DiScala and Glimm 33 consider an adaptive trial design with a survival endpoint, in which treatment selection is based on a more rapidly observed event. When analysing follow-up data on subjects who have already contributed to a decision about treatment choice, there is a danger of type I error inflation 34 but the methods of Jenkins *et al*. 35 and Irle and Schäfer 36 can be used to avoid this problem.

In section 6, we considered the case where ‘treatments’ represent dose levels and a dose–response model may be used. The smaller risk of safety problems at lower dose levels motivates the decision in the MCP-mod procedure of Bretz *et al*. 23 to select the lowest dose that produces a specified treatment effect, even when safety responses are not considered directly. Some authors have considered treatment selection and testing based on both efficacy and safety data: Liu and Pledger 15 refer to safety outcomes when deciding on the treatment to take forward from the first stage of a seamless phase II/III design; König *et al*. 37 and Kimani *et al*. 38 propose further procedures for this case. If a model for efficacy, and possibly safety, is specified and the benefits of demonstrating a new treatment to be effective are quantified, the question of how best to design two (or more) phases of a drug development programme can be clearly stated. The problem is challenging, even without the combination of data across stages of a seamless design. This is an area of considerable current activity that is starting to produce important insights; see, for example, 39,40.

## Appendix A

Connection between optimal data combination rules and parameter estimates for a linear model

We consider the case of Section 5.1 where ***θ*** is a permutation of (*γ*,…,*γ*,1)*δ* for known *γ*∈(0,1). Let , *i* = 0,1,…,*K*, denote the first stage sample means on the control and *K* experimental treatments, so the estimated treatment effects in stage 1 are , *i* = 1,…,*K*. For simplicity, suppose  is the largest stage 1 estimate and treatment *i*⋆=*K* is compared against control in stage 2.

The optimal decision rule (14) in Section 5.1 is based on the likelihood ratio of the combined stage 1 and stage 2 data under ***θ*** = ***ξ***_*i*⋆_=(*γ*,…,*γ*,1)*δ* and ***θ*** = **0**, and this can be written as the product of separate terms for stage 1 and stage 2 data. The stage 1 estimates  follow a normal linear model with a single unknown parameter *δ*; also, given *i*⋆=*K*, the stage 2 estimate  is normally distributed with mean *δ*. Let  and  be the maximum likelihood estimates of *δ* based on stages 1 and 2 data, respectively, with variances  and . Standard algebra shows that the log likelihood ratio between ***θ*** = ***ξ***_*i*⋆_ and ***θ*** = **0** for stage 1 data is a constant plus , and for stage 2, data it is a constant plus . Combining these terms, we find the log likelihood ratio based on the stage 1 and stage 2 data together is an increasing function of

a multiple of the maximum likelihood estimate of *δ* for the pooled stages 1 and 2 data. It follows that the first-stage estimates  contribute to the optimal decision rule with weights proportional to their weights in , the estimate of *δ* obtained by fitting a normal linear model to .

At this point, it helps to represent the stage 1 data as the sample means , *i* = 0,1,…,*K*, on the control and *K* experimental treatments. In the case we are considering, ***θ*** = (*γ*,…,*γ*,1)*δ* and  follow a linear regression model with , where *x*_0_=0, *x*_1_=…=*x*_*K* − 1_=*γ* and *x*_*K*_=1. The estimate  is a linear combination of  with weights summing to zero, so

and we see  contribute to  with the same weights as . If *γ* = 0, so ***θ*** = (0,…,0,1)*δ*, it is straightforward to show

in agreement with the contributions of  in the decision rule (18), which can be regarded as a test of *H*_0_: *δ* = 0. If *γ* = 0.5,  make no contribution to the estimate of the slope *δ* in the linear regression  and so have zero weight in , in keeping with their absence from the TSE decision rule, which is optimal in this case. For other values of *γ*, inspection of the linear regression model shows that  contribute to  with negative weights if *γ* < 0.5 and with positive weights if *γ* > 0.5, which agrees with the pattern of weights for unselected first-stage treatments in the data combination rule (18).

## Supporting Information

Supporting info item

## References

[b1] Thall PF, Simon R, Ellenberg SS (1988). Two-stage selection and testing designs for comparative clinical trials. Biometrika.

[b2] Sampson AR, Sill MW (2005). Drop-the-losers design: normal case. Biometrical Journal.

[b3] Bretz F, Schmidli H, König F, Racine A, Maurer W (2006). Confirmatory seamless phase II/III clinical trials with hypotheses selection at interim: general concepts (with discussion). Biometrical Journal.

[b4] Schmidli H, Bretz F, Racine A, Maurer W (2006). Confirmatory seamless phase II/III clinical trials with hypotheses selection at interim: applications and practical considerations. Biometrical Journal.

[b5] Marcus R, Peritz E, Gabriel KR (1976). On closed testing procedures with special reference to ordered analysis of variance. Biometrika.

[b6] Bauer P, Köhne K (1994). Evaluation of experiments with adaptive interim analyses. Biometrics.

[b7] Jennison C, Turnbull BW (2006). Discussion of papers on “Confirmatory seamless phase II/III clinical trials with hypotheses selection at interim”. Biometrical Journal.

[b8] Bauer P, Kieser M (1999). Combining different phases in the development of medical treatments within a single trial. Statistics in Medicine.

[b9] Fisher RA (1932). Statistical Methods for Research Workers.

[b10] Mosteller F, Bush RR, Lindsey G (1954). Selected quantitative techniques. Handbook of Social Psychology.

[b11] Lehmacher W, Wassmer G (1999). Adaptive sample size calculations in group sequential trials. Biometrics.

[b12] Simes RJ (1986). An improved Bonferroni procedure for multiple tests of significance. Biometrika.

[b13] Dunnett CW (1955). A multiple comparison procedure for comparing several treatments with a control. Journal of the American Statistical Association.

[b14] Sarkar SK, Chang CK (1997). The Simes method for multiple hypothesis testing with positively dependent test statistics. Journal of the American Statistical Association.

[b15] Liu Q, Pledger GW (2005). Phase 2 and 3 combination designs to accelerate drug development. Journal of the American Statistical Association.

[b16] Jennison C, Turnbull BW (2007). Adaptive seamless designs: selection and prospective testing of hypotheses. Journal of Biopharmaceutical Statistics.

[b17] Eales JD, Jennison C (1992). An improved method for deriving optimal one-sided group sequential tests. Biometrika.

[b18] Barber S, Jennison C (2002). Optimal asymmetric one-sided group sequential tests. Biometrika.

[b19] Banerjee A, Tsiatis AA (2006). Adaptive two-stage designs in phase II clinical trials. Statistics in Medicine.

[b20] Öhrn F, Jennison C (2010). Optimal group sequential designs for simultaneous testing of superiority and non-inferiority. Statistics in Medicine.

[b21] Hampson LV, Jennison C (2013). Group sequential tests for delayed responses (with discussion). Journal of the Royal Statistical Society B.

[b22] Robbins H, Monro S (1951). A stochastic approximation method. The Annals of Mathematical Statistics.

[b23] Bretz F, Pinheiro JC, Branson M (2005). Combining multiple comparisons and modeling techniques in dose-response studies. Biometrics.

[b24] Bretz F, Strassburger K, Maurer W (2005). Dicussion of “Drop-the-losers design: Normal case”. Biometrical Journal.

[b25] Bischoff W, Miller F (2005). Adaptive two-stage test procedures to find the best treatment in clinical trials. Biometrika.

[b26] Stallard N, Todd S (2003). Sequential designs for phase III clinical trials incorporating treatment selection. Statistics in Medicine.

[b27] Jennison C, Turnbull BW (2000). Group Sequential Methods with Applications to Clinical Trials.

[b28] Kelly PJ, Stallard N, Todd S (2005). An adaptive group sequential design for phase II/III clinical trials that select a single treatment from several. Journal of Biopharmaceutical Statistics.

[b29] Stallard N, Friede T (2008). A group-sequential design for clinical trials with treatment selection. Statistics in Medicine.

[b30] Stallard N (2010). A confirmatory seamless phase II/III clinical trial design incorporating short-term endpoint information. Statistics in Medicine.

[b31] Magirr D, Jaki T, Whitehead J (2012). A generalized Dunnett test for multi-arm multi-stage clinical studies with treatment selection. Biometrika.

[b32] Wason J, Jaki T (2012). Optimal design of multi-arm multi-stage trials. Statistics in Medicine.

[b33] Di Scala L, Glimm E (2011). Time-to-event analysis with treatment arm selection at interim. Statistics in Medicine.

[b34] Bauer P, Posch M (2004). Modification of the sample size and the schedule of interim analyses in survival trials based on data inspections (letter to the editor). Statistics in Medicine.

[b35] Jenkins M, Stone A, Jennison C (2011). An adaptive seamless phase II/III design for oncology trials with subpopulation selection using correlated survival endpoints. Pharmaceutical Statistics.

[b36] Irle S, Schäfer H (2012). Interim design modifications in time-to-event studies. Journal of the American Statistical Association.

[b37] König F, Bauer P, Brannath W (2006). An adaptive hierarchical test procedure for selecting safe and efficient treatments. Biometrical Journal.

[b38] Kimani PK, Stallard N, Hutton JL (2009). Dose selection in seamless phase II/III clinical trials based on efficacy and safety. Statistics in Medicine.

[b39] Patel N, Bolognese J, Chuang-Stein C, Hewitt D, Gammaitoni A, Pinheiro J (2012). Designing phase 2 trials based on program-level considerations: a case study for neuropathic pain. Drug Information Journal.

[b40] Marchenko O, Miller J, Parke T, Perovozskaya I, Quian J, Wang Y (2013). Improving oncology clinical program by use of innovative designs and comparing them by simulations. Therapeutic Innovation and Regulatory Science.

