# Supplementary material for: Optimizing the data combination rule for seamless phase II/III clinical trials
Source: Stat Med. 2014 Oct 15;34(1):39–58. doi: 10.1002/sim.6316 (PMC4288236; doi:10.1002/sim.6316)
Supplement: Supplementary file 1 — Supporting info item [file sim0034-0039-sd1.pdf]

Web-based Supporting Materials for “Optimising the Data Combination Rule for Seamless Phase II/III Clinical Trials”

Lisa V. Hampson[†] and Christopher Jennison[‡]

[†]Department of Mathematics and Statistics,
Fylde College,
Lancaster University,
Lancaster LA1 4YG,
UK.

[‡]Department of Mathematical Sciences,
University of Bath,
Bath BA2 7AY,
UK.

June 24, 2014

Maintaining strong control of the FWER for seamless Phase II/III designs that use combination tests to combine data across stages of a trial

Following the notation and terminology of Section 3 of the main manuscript, we consider closed testing procedures that use combination rules to combine p-values in the test of each intersection hypothesis. In the study design we consider, only $H_{0,i^\star}$ can be rejected at the end of Stage 2 and this happens if every intersection hypothesis $H_{0,I}$ for which $i^\star \in I$ is rejected at level α. Then, the "BK inverse χ^2" decision rule rejects $H_{0,i^\star}$ if

$$\min_{\{I \subseteq \mathcal{S}:\, i^\star \in I\}}\{-\log(P_{1,I}) - \log(P_{2,I})\} \;>\; 0.5\,\chi^2_{4,1-\alpha}. \tag{1}$$

The "BK inverse normal" decision rule rejects $H_{0,i^\star}$ if

$$\min_{\{I \subseteq \mathcal{S}:\, i^\star \in I\}}\{w_1\, Z_{1,I} + w_2\, Z_{2,I}\} \;>\; \Phi^{-1}(1-\alpha). \tag{2}$$

In our investigations of the above decision rules, we consider two approaches to defining the p-values for intersection hypotheses. Suppose $H_{0,I}$ is the intersection of m simple null hypotheses and denote the Stage 1 p-values of these hypotheses, arranged in ascending order, by $P_{1,(1)}, \ldots, P_{1,(m)}$. Using Simes' [1] method, the p-value for testing $H_{0,I}$ is

$$P_{1,I} = \min_{k=1,\ldots,m}\{m\, P_{1,(k)}/k\} \tag{3}$$

and this is valid, but conservative, for p-values arising from multiple comparisons with a common control [2]. In applying the rules (1) and (2), we only need to consider hypotheses $H_{0,I}$ where $i^\star \in I$. Then, since the best performing treatment is selected at the end of Stage 1, $P_{1,(1)} = P_{1,i^\star}$ and a simple Bonferroni adjustment would give $P_{1,I} = m\, P_{1,i^\star} = m\, P_{1,(1)}$. If other treatments also perform well in Stage 1, a low value of $P_{1,(k)}/k$ for some $k > 1$ can reduce $P_{1,I}$ in (3). Thus Simes' method 'borrows strength' from results on treatments other than $i^\star$.

Bretz et al. [3] use Dunnett [4] p-values for intersection hypotheses. These are appropriate for multiple comparisons with a control where the joint distribution of the z-statistics Z_i for hypotheses $H_{0,i}$, $i \in I$, is multivariate normal with $\mathrm{cov}(Z_i, Z_{i'}) = 0.5$ for $i \neq i'$. Let $z^\star$ be the maximum of the m observed z-values for hypotheses $H_{0,i}$, $i \in I$. Then, the Dunnett p-value is

$$\mathrm{pr}\{\max_{i \in I} Z_i \;>\; z^\star\} \tag{4}$$

calculated under the above multivariate normal distribution with each $Z_i \sim N(0,1)$, the least favourable configuration in $H_{0,I}$. Since $i^\star$ is associated with the largest $\hat{\theta}_{1,i}$ in Stage 1, the probability (4) is maximised when $I = S$, so only this case need be considered in taking the minimum in (1) or (2).

Testing intersection hypotheses in Stage 2 is a simple task. The only comparison available in Stage 2 is between treatment $i^\star$ and the control, so we set $P_{2,I} = P_{2,i^\star}$ for all I containing $i^\star$.

To adjust for the conservatism that may arise due to the possibility of stopping for futility in Stage 1 of the trial and using Simes' method to define p-values for intersection hypotheses, in our investigations of decision rules we have adjusted the critical values on the right hand sides of (1) and (2) so that the FWER is α

under $\boldsymbol{\theta} = \mathbf{0}$. We argue that this is a sufficient condition to ensure strong control of the FWER.

In proving this claim, it is easy to see that the FWER is still strongly controlled when all null hypotheses $H_{0,1}, \ldots, H_{0,K}$ are true, so $\theta_i \leq 0$ for all $i = 1, \ldots, K$. Decreasing one of these θ_i from zero to $-\delta_i$, say, has the same effect on the distribution of Stage 1 estimates as subtracting a constant δ_i from the value of $\hat{\theta}_{1,i}$ observed when $\theta_i = 0$. Using either Simes' or Dunnett's method, a deterministic reduction in $\hat{\theta}_{1,i}$ leads to lower values of $-\log(P_{1,I})$ for sets I containing $i^\star$ in (1) and lower values of $Z_{1,I}$ in (2). Moreover, the distribution of $P_{2,i^\star}$ is stochastically larger and that of $Z_{2,i^\star}$ stochastically smaller when $\theta_{i^\star} < 0$ than when $\theta_{i^\star} = 0$. It follows that the highest familywise error probability when $H_{0,1}, \ldots, H_{0,K}$ are all true occurs at $\boldsymbol{\theta} = \mathbf{0}$.

Proving that tests with adjusted critical values in (1) and (2) strongly control the FWER when some $\theta_i > 0$ is more difficult. Jennison & Turnbull [5] show that increasing a θ_i above zero reduces the FWER of the TSE decision rule. The same argument can be applied to a combination test using Dunnett's test for intersection hypotheses. Suppose $\theta_i > 0$, then $H_{0,i}$ is false, so deciding in favour of treatment i is no longer a type I error; moreover, if a treatment $i^\star$ with $\theta_{i^\star} \leq 0$ is selected, the value of $\hat{\theta}_{1,i}$ has no bearing on $P_{1,I}$ for sets $I \ni i^\star$ so the probability of rejecting any true hypothesis does not increase. If, instead, Simes' method is used to test intersection hypotheses, taking $\theta_i > 0$ still makes $H_{0,i}$ false, so deciding in favour of treatment i is no longer a type I error. However, a low p-value for treatment i may appear as a term in (3) with $k > 1$ and reduce the p-value for an intersection hypothesis involving a selected treatment $i^\star \neq i$. We expect the reduction in the number of true null hypotheses and the increased probability that a treatment with $\theta_i > 0$ is selected after Stage 1 to have the dominant effect, so that increasing a θ_i above zero reduces the overall probability of a familywise error. In all of the empirical examples that we have so far considered, we have found that applying these lower critical values controls the FWER with some conservatism. We emphasise that if the unadjusted critical values, as stated in (1) and (2), are used then strong control of the FWER is guaranteed.

The seamless designs we have considered stipulate that treatment $i^\star$, with the maximum $\hat{\theta}_{1,i}$, is taken forward to Stage 2. However, greater flexibility is possible. This is important as a treatment with a lower value of $\hat{\theta}_{1,i}$ might be chosen for safety considerations, either because of adverse events observed in Stage 1 or perceived safety risks for a higher dose or more aggressive treatment. All of the decision rules that we have considered (conventional, TSE, "BK inverse χ^2", "BK inverse normal") can be viewed as closed testing procedures (Jennison & Turnbull [5] show this is true for the TSE method) and this offers a useful framework for more flexible implementations, enhancing power where possible while continuing to protect the FWER. However, if the methods are to be used more flexibly, we would recommend use of the original critical values stated in (1) and (2) unless it can be proved unequivocally that the FWER is fully protected.

References

[1] Simes, RJ. An improved Bonferroni procedure for multiple tests of significance.

Biometrika 1986; **73**:751–754.

[2] Sarkar, SK, Chang, C-K. The Simes method for multiple hypothesis testing with positively dependent test statistics. *Journal of the American Statistical Association* 1997; **92**:1601–1608.

[3] Bretz F, Schmidli, H, König, F, Racine, A, Maurer, W. Confirmatory seamless phase II/III clinical trials with hypotheses selection at interim: General concepts (with discussion). *Biometrical Journal* 2006; **48**:623–634.

[4] Dunnett, CW. A multiple comparison procedure for comparing several treatments with a control. *Journal of the American Statistical Association* 1955; **50**:1096–1121.

[5] Jennison C, Turnbull BW. Discussion of papers on “Confirmatory seamless phase II/III clinical trials with hypotheses selection at interim”. *Biometrical Journal* 2006; **48**:650 – 655.
